# Type 2 diabetes and healthcare resource utilisation in the Kingdom of Bahrain

**DOI:** 10.1186/s12913-019-4795-5

**Published:** 2019-12-05

**Authors:** Rabha AbdulAziz Salman, Adel Salman AlSayyad, Craig Ludwig

**Affiliations:** 1 Ministry of Health, Manama, Kingdom of Bahrain; 2 Associate professor, Arabian Gulf University, Manama, Kingdom of Bahrain; 3 Last Mile, Holte, Denmark

**Keywords:** Type 2 diabetes, Healthcare resource utilisation, Direct costs, Indirect costs, Micro- and macrovascular complications

## Abstract

**Background:**

Type 2 diabetes is a growing health challenge in the Kingdom of Bahrain, and the disease exerts significant pressure on the healthcare system. The aim of this study was to assess the annual costs and understand the drivers of those costs in the country.

**Methods:**

A sample of 628 patients diagnosed with type 2 diabetes were randomly selected from primary healthcare diabetes clinics, and the direct medical and indirect costs due to type 2 diabetes were analysed for a one-year period. The study used patients’ medical records, interviews and standardised frequency questionnaires to obtain data on demographic and clinical characteristics, complication status, treatment profile, healthcare resource utilisation and absenteeism due to diabetes. The indirect costs were estimated by using the human capital approach. The direct medical and indirect costs attributable to type 2 diabetes were extrapolated to the type 2 diabetes population in Bahrain.

**Results:**

In 2015, the total direct medical cost of type 2 diabetes was 104.7 million Bahraini dinars (BHD), or 277.9 million US dollars (USD), and the average unit cost per person with type 2 diabetes (1162 BHD, or 3084 USD) was more than three times higher than for a person without the condition (372 BHD, or 987 USD). The healthcare costs for patients with both micro- and macrovascular complications were more than three times higher than for patients without complications. Thus, 9% of the patients consumed 21% of the treatment costs due to complications. Complications often lead to hospital admission, and 20% of the patients consumed almost 60% of the healthcare costs attributable to type 2 diabetes due to hospital admissions. The indirect cost due to absenteeism was 1.23 million BHD (3.26 million USD).

**Conclusion:**

Type 2 diabetes exerts significant pressure on Bahrain’s healthcare system – primarily due to costly diabetes-related complications. It is therefore important to optimise the management and control of type 2 diabetes, thereby reducing the risk of disabling and expensive complications.

## Background

Diabetes is a serious disease and a growing public health challenge in every part of the world [1]. The International Diabetes Federation (IDF) estimates that currently 425 million adults have diabetes globally and expects this number to increase to 629 million by 2045 [2].

There are two primary forms of diabetes, which are more often than not grouped together, but the causes and costs of which are different. Type 1 diabetes is an autoimmune disease [2] and is estimated to account for 10–15% of cases [3–5]. People with type 1 diabetes require insulin to survive [2]. Despite being largely preventable, type 2 diabetes (T2D) accounts for about 90% of cases [6–8]. In T2D, the body is able to produce insulin but becomes resistant so that the insulin is ineffective. Over time, insulin levels may subsequently become insufficient. Both the insulin resistance and deficiency lead to high blood glucose levels [2]. If blood glucose levels continue to rise due to the diabetes not being well controlled, the risk of serious and morbid complications increases [9]. For example, diabetes is the primary cause of renal failure, amputation and vision loss [10].

The healthcare costs of diabetes are substantial and include expenditure on resources used for treating the condition, such as outpatient consultations, diagnostic testing, medications, emergency visits and inpatient procedures and care [11]. Globally, 727 billion USD, or 12% of direct healthcare expenditures, was spent on diabetes in 2017, and this figure is projected to reach 776 billion USD by 2045 [2].

From other countries where research into the economic burden of diabetes exists, it is evident that a substantial portion of direct healthcare expenditure is driven by complications related to diabetes. In the US, the American Diabetes Association (ADA) estimated that the total cost of diagnosed diabetes in 2017 was 327 billion USD, including 237 billion in direct medical costs and 90 billion in reduced productivity [12]. Prescription medications for the treatment of diabetes-related conditions like hypertension and cardiovascular disease accounted for 30% of the total direct medical expenditure while another 30% was due to admissions [12].

In France, Charbonnel et al. found that the average medical expenditure for a person with T2D was 6506 EUR (7205 USD) in 2013, accounting for 8.5 billion EUR (9.4 billion USD) of the country’s medical costs [13]. Furthermore, they identified the highest individual direct costs incurred were related to admissions at 33.2% of total costs [13]. Similarly, in Italy, a study from 2014 noted that the most important contributor to direct costs was admissions, which accounted for almost 53% of the total 9.6 billion EUR [14]. In the UAE, using a macro cost approach, Al-Maskari et al. estimated the annual direct cost of diabetes to be around 1605 USD for patients without complications, with the values increasing significantly with the addition of complications [15].

Though, in general, lower than the direct costs, the indirect costs associated with diabetes – the value of the loss in productivity due to morbidity and mortality – are also considerable [11, 16, 17]. However, indirect costs are challenging to estimate, and the variation across studies is high.

The Gulf Cooperation Council (GCC) countries (Bahrain, Kuwait, Oman, Qatar, Saudi Arabia and the United Arab Emirates) are part of the Middle East and North Africa (MENA) region, which currently has the world’s second-highest age-adjusted comparative diabetes prevalence: 10.8% [2]. If current trends continue, the number of people with diabetes in the region is expected to increase by 72% by 2045 [2]. The main drivers for the steep rise in these countries are major demographic and socioeconomic transitions and parallel shifts in culture, lifestyle and dietary habits, together with increased life expectancy and genetic predisposition to T2D [18]. In addition, it has been shown that more than 70% of people with diabetes in MENA countries have poorly controlled diabetes [19], leading to high rates of complications. Thus, the pressure on countries’ healthcare budgets in this region is high and, proportionally, the region has the highest percentage of healthcare budget spent on diabetes, at close to 17% in 2017 [2]. The diabetes cost in the MENA region was estimated at 21 billion USD in 2017 and is expected to increase to 36 billion USD by 2045 [2].

In Bahrain, a national health survey revealed an overall prevalence of T2D of 14.3% among Bahraini nationals in the age group 20–64 [20] – the age group considered to be the most important for social and economic productivity. According to the IDF, diabetes healthcare expenditure in Bahrain was 86 BHD (227 million USD) in 2015, translating to a unit cost per person with diabetes of 555 BHD (1473.50 USD) [21]. This is a more than sixfold increase in just 12 years, up from 14 million BHD (37 million USD) in 2003 [21, 22]. However, IDF estimates of diabetes costs in GCC countries have been shown to underestimate the actual costs [23], and the impact of diabetes on Bahrain’s healthcare budget and overall economy is thought to be far greater than the figures reported by the IDF. Back in 2017, the IDF significantly increased its estimate of diabetes-related healthcare expenditure in Bahrain to 110 million BHD (292 million USD), or a unit cost per person with diabetes of 667 BHD (1769.9 USD) [2].

Research into the economic burden of diabetes, and specifically that of T2D in Bahrain and other GCC countries, is lacking. Such research is required to guide decision-makers towards initiating disease prevention and management strategies that can prevent future healthcare budgets ‘drowning’ under the pressure of diabetes. To our knowledge, this is the first cost-of-illness study estimating the healthcare costs of T2D in Bahrain to include the costs of direct management of diabetes and diabetes-related micro- and macrovascular complications as well as absenteeism due to T2D as a proxy for indirect costs.

## Methods

### Aim, design and setting

The study used a prevalence-based cost-of-illness approach, and data were collected between March 2011 and December 2012 to cover a one-year period for each recruited patient. Patient recruitment and data collection were performed in primary healthcare diabetes clinics.

The Kingdom of Bahrain has a comprehensive healthcare system organised around a network of primary health centres, secondary care hospitals and tertiary care centres. Treatment and care for people with T2D are primarily managed through 28 primary health centres distributed across the five regions of the country. The centres are geographically located so that there is a health centre within a 20-min drive of all residents’ homes. Each centre has laboratory facilities, a theatre for minor surgery, general and diabetes clinics, maternal and child health and physiotherapy departments, primary care physicians, diabetologists, general and diabetes nurses, health educators, social workers and physiotherapists.

All health centres use electronic files and are networked with each other and with the main hospitals. Diabetes clinics are run by a diabetologist and a diabetes nurse, and they are where newly diagnosed and/or uncontrolled diabetes cases are referred for assessment, management and education. Diabetologists in these clinics can prescribe every range of antidiabetic medicine in addition to glucometers and strips. Patients are then referred back to their general practitioner (GP) in the same health centre for routine follow-up.

Generally, only very advanced cases of T2D – those requiring specialist consultation – are referred to a secondary care facility. Other types of diabetes, such as type 1 diabetes and gestational diabetes, are seen at secondary healthcare facilities.

In 2012, the year of data collection, healthcare services were provided free of charge to Bahraini nationals, and some people were covered by insurance through their employers.

### Characteristics of participants or description of materials

Participants were selected using multi-stage stratified cluster sampling. Nine primary healthcare diabetes clinics were randomly selected, and the sample size was divided among these. The sample size for each clinic was proportional to the catchment area. Participants were selected by systematic random sampling (every 10th patient) from the clinics’ registries. The study participants included male and female Bahraini nationals with T2D between the ages of 20 and 64.

The required sample size for a prevalence study of T2D was calculated as:

$$ N=\frac{Fp\left(1-p\right)}{d^2} $$

where N is the sample size, F is 10.51 (power of 90% and significance of 95%), P is 17.5% (expected proportion in population based on previous studies is 15–20%) and d is 2.5% (smallest effect of interest). Furthermore, taking into account a non-response rate of 25% and a prevalence of T2D of 15–20%, the required sample size is 455–607 subjects. Complete data on 628 people with T2D were obtained and analysed. Interviews and a standardised frequency questionnaire, specifically developed for this study (Additional file 1), were completed to obtain data on demographics, healthcare resource utilisation, healthcare provider, health insurance status and impact on work in the form of absenteeism due to T2D in the previous 12 months. Clinical information regarding T2D, its complications and the treatment profile of participants was based on the patients’ medical records. Diabetes-related complications were grouped by microvascular complications (nephropathy, peripheral neuropathy and retinopathy) and by macrovascular complications (ischaemic heart disease, cerebrovascular accidents or transient ischaemic attacks and peripheral vascular disease).

### Data analysis

The direct medical costs related to T2D were classified into seven resource categories: admissions, procedures (cardiac catheterisation, laser treatment and cataract surgery), outpatient (OP) visits (primary healthcare and secondary healthcare), laboratory tests, oral antidiabetic medicine, other oral medicine used to treat comorbidities, insulin and other injectables, and self-monitoring (glucometer device and strips). A bottom-up approach was used to estimate the total medical cost for each patient as the unit cost for each resource used multiplied by the total resources used. The cost of each resource item was obtained from a 2015 internal document provided by the Supportive Services Directorate of Bahrain’s Ministry of Health (MOH) [24]. Thus, the cost calculations relate to the year 2015. Healthcare services provided to people with diabetes remained unaltered between 2012 and 2015. The average cost for each patient per year was applied to all people with T2D in Bahrain. The direct medical costs were calculated for people with T2D without complications, for people with T2D and microvascular complications, for people with T2D and macrovascular complications, and for people with T2D and both micro- and macrovascular complications. The indirect costs of T2D were approximated by productivity losses due to absenteeism related to T2D. We used the human capital approach, where days absent were multiplied by average earnings per capita per day [25]. According to the Labour Market Regulatory Authority of Bahrain, the average daily wage was 24.9 BHD (66.1 USD^1^) in 2015 [26].

The overall number of people with T2D in Bahrain was used to extrapolate the cost estimates for the study sample to a population estimate. The number of people with T2D in Bahrain was estimated to be 90,110, based on a population size of 1.3 million in 2015 and a prevalence of T2D of 14.3% among Bahrainis [20] and of 6.2% among non-Bahrainis [27] for the 20–64 age group. T2D costs were assumed to be similar for Bahrainis and non-Bahrainis.

The research was approved by the MOH, and an informed consent for agreement to participate in the study was obtained from all study participants.

### Analysis

Descriptive statistics were used to describe continuous variables, and frequencies were used to describe categorical variables. 

## Results

### Demographic and clinical characteristics

Table 1 shows the demographic characteristics of the participants in the study. Almost two-thirds (61.3%) were women, one-quarter (23.4%) were illiterate and the majority (65.6%) were aged between 40 and 59.

**Table 1:** Demographic characteristics of study participants (*N* = 628)

Patient characteristics		N	%
Gender	Male	243	38.7
	Female	385	61.3
Education level	Illiterate	146	23.4
	Primary school	111	17.8
	Intermediate school	105	16.8
	Secondary school	157	25.2
	Higher education	105	16.8
Age	20–39	52	8.3
	40–59	412	65.6
	60–64	164	26.1

Table 2 illustrates the clinical characteristics, healthcare provider and health insurance status of the people in the sample. The average duration of T2D was 8.1 ± 6.7 years (mean ± sd), and 36.7% of the participants had been diagnosed within the previous five years. The majority (80.3%) of participants had a family history of T2D, and 80.7% were non-smokers. The average BMI was 31.3 ± 7.3 (mean ± sd), and 56.5% of the participants had obesity (BMI ≥ 30). Around half (53.3%) of the participants had hypertension, and almost all (90.6%) had dyslipidaemia. Good blood glucose control, which guidelines consider to be glycated haemoglobin (HbA_1c_) below 7% for T2D [2, 28], was achieved by half (52.6%) of the participants. Conversely, 47.4% were not in good control, and a remarkable proportion of the participants (20.9%) had an HbA_1c_ above 9%. The majority of the participants had been seen at a primary care centre (88.5%), and 26.8% had been seen at a public hospital. Almost all (91.1%) of the participants had no health insurance.

**Table 2:** Clinical characteristics, healthcare provider and health insurance status of study participants (*N* = 628 unless otherwise stated)

Patient characteristics		N	%
Duration of T2D (*N* = 619)	< 5 years	227	36.7
	5–10 years	163	26.3
	11–20 years	173	27.9
	> 20 years	56	9.0
Family history of T2D	Yes	504	80.3
	No	124	19.7
BMI (*N* = 621)	< 25	102	16.4
	25–29	168	27.1
	≥ 30	351	56.5
Hypertension	Yes	335	53.3
	No	293	46.7
Dyslipidaemia	Yes	569	90.6
	No	59	9.4
HbA_1c_ (*N* = 622)	< 7%	327	52.6
	7–8%	95	15.3
	8–9%	72	11.6
	> 9%	128	20.6
Smoking status (*N* = 623)	Smoker	82	13.2
	Non-smoker	503	80.7
	Ex-smoker	38	6.1
Healthcare provider	Public hospital	168	26.8
	Primary care centre	556	88.5
	Private hospital	60	9.6
	Private clinic	28	4.5
Health insurance	Yes	56	8.9
	No	572	91.1

*T2D* type 2 diabetes, *BMI* body mass index, *HbA*_*1c*_ glycated haemoglobin

### Complication status of study participants

Table 3 shows the complication status of the participants. The results showed that diabetes-related complications were common: almost half of the participants (43.4%) had at least one complication, 38.5% had microvascular complications, 14.2% had macrovascular complications, 9.2% had both micro- and macrovascular complications, and 34.2% had either micro- or macrovascular complications.

**Table 3:** Complication status of study participants (*N* = 628)

Complication status	N	%
No complications	355	56.6
Microvascular complications	242	38.5
Macrovascular complications	89	14.2
Either micro- or macrovascular complications	215	34.2
Both micro- and macrovascular complications	58	9.2

### Healthcare costs attributable to T2D according to resource categories

**Fig. 1:**
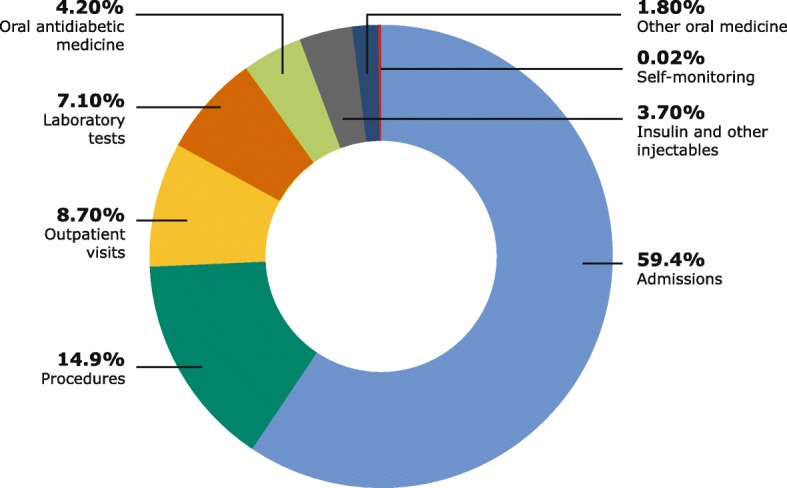
Relative distribution of direct medical costs attributable to T2D according to resource categories

Table 4 shows the use of main resource categories by participants, Table 5 demonstrates the costs of these resources extrapolated to the entire T2D population living in Bahrain and Fig. 1 illustrates the relative distribution of cost by resource.

**Table 4:** Use of main resource categories among study participants (*N* = 628)

Main resources		N	%
Admissions	Yes	121	19.3
	No	507	80.7
Procedures	Yes	77	12.3
	No	551	87.7
Outpatient visits	Yes	554	88.2
	No	74	11.8
Laboratory tests	Yes	554	88.2
	No	74	11.8
Oral antidiabetic medicine	Yes	561	89.3
	No	67	10.7
Other oral medicine to treat comorbidities	Yes	447	71.2
	No	181	18.8
Insulin and other injectables	Yes	148	23.6
	No	480	76.4
Self-monitoring	Yes	187	29.8
	No	441	70.2

Procedures covers cardiac catheterisation, laser treatment and cataract surgery; Outpatient visits covers primary and secondary care; Other oral medicine covers blood pressure-lowering medicine and lipid-lowering medicine; Self-monitoring covers lancets and strips

**Table 5:** Annual direct medical costs attributable to T2D according to main resource categories among study participants in Bahrain

	For each patient	Total population	For each patient	Total population
	BHD (95% CI)	USD (95% CI)	Million BHD (95% CI)	Million USD (95% CI)
Admissions	690 (485–895)	1831 (1287–2374)	62.2 (43.7–80.6)	165.0 (116.0–214.0)
Procedures	173 (122–225)	460 (323–598)	15.6 (11.0–20.3)	41.5 (29.1–53.9)
Outpatient visits	101 (94–108)	269 (250–288)	9.1 (8.5–9.8)	24.2 (22.5–26.0)
Laboratory tests	82 (75–89)	218 (200–235)	7.4 (6.8–8.0)	19.6 (18.0–21.2)
Oral antidiabetic medicine	49 (46–51)	130 (122–137)	4.4 (4.2–4.6)	11.7 (11.0–12.3)
Insulin and other injectables	42 (34–51)	113 (91–134)	3.8 (3.1–4.6)	10.2 (8.2–12.1)
Other oral medicine	21 (10–24)	57 (49–65)	1.9 (1.7–2.2)	5.1 (4.4–5.8)
Self-monitoring	2 (945–1379)	6 (4–9)	0.2 (0.13–0.3)	0.56 (0.34–0.78)
Total costs^a^	1162 (945–1379)	3084 (2507–3659)	104.7 (85.1–124.2)	277.8 (225.9–329.7)

Self-monitoring covers lancets and strips; Oral medicine covers oral antidiabetic medicine, blood pressure-lowering medicine and lipid-lowering medicine; Procedures covers cardiac catheterisation, laser treatment and cataract surgery; M: million

^a^ Due to rounding, some totals may not correspond to the sum of the separate figures

Of the 628 people in the sample, almost one in five (19.3%) were admitted to hospital during this one-year period. Annual costs due to admissions amounted to 690 BHD (1831 USD) for each patient and 62.2 million BHD (165.1 million USD) for all people with T2D living in Bahrain and were by far the largest expenditure, accounting for 59.4% of the total costs of T2D. Procedures which covered cardiac catheterisation, laser treatment and cataract surgery were performed on 12.3% of the participants. The annual cost of the procedures amounted to 173 BHD (459 USD) for each patient and 15.6 million BHD (41.4 million USD) for the entire T2D population, constituting 14.9% of the total costs. 88.2% of the participants were outpatients (OP) (GP and secondary care visits), and laboratory tests were conducted on the same share of participants. The annual cost of OP visits was 101 BHD (268 USD), and the cost of laboratory tests was 82 BHD (218 USD) for each patient, corresponding to 9.1 and 7.4 million BHD respectively (24.2 and 19.6 million USD) for the T2D population. This represented 8.7 and 7.1% respectively of the total cost. Almost one in four participants (23.6%) used insulin and other injectables, 89.3% used oral antidiabetic medicine and 71.2% used oral medicine to treat comorbidities such as hypertension and hyperlipidaemia. The relative cost of all medicines combined was below 10%. Insulin and other injectable antidiabetic medicine accounted for 3.7%, or 42 BHD (111 USD), for each patient, and 3.8 million BHD (10.1 million USD) for the T2D population annually. The annual cost fraction of oral antidiabetic medicine was 4.2% and accounted for 49 BHD (130 USD) for each patient and 4.4 million BHD (11.7 million USD) for all people in Bahrain with T2D. Other oral medicines, including blood pressure- and lipid-lowering agents, made up 1.8% of annual T2D costs: 21 BHD (56 USD) for each patient and 1.9 million BHD (5.0 million USD) for all the people with T2D in Bahrain. Of the 628 participants, 29.8% monitored their blood glucose. The annual cost of lancets and strips for self-monitoring of blood glucose was 2 BHD (5 USD) for each patient and 0.2 million BHD (0.5 million USD) for all the people with T2D in Bahrain, accounting for a small fraction (0.2%) of the total costs.

### Healthcare costs attributable to T2D according to complication status

**Fig. 2:**
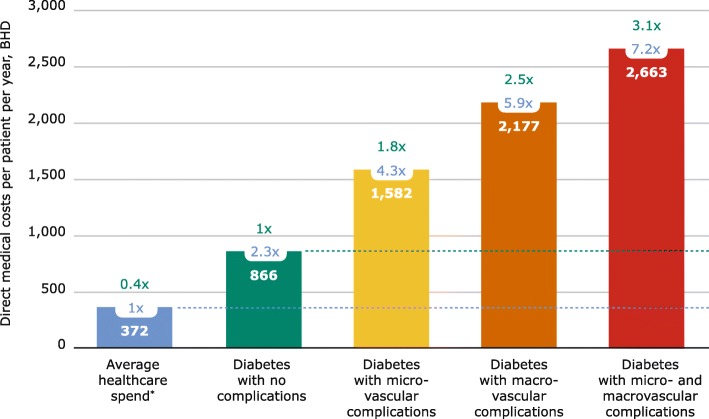
Direct medical costs according to complication status among study participants (*N*=628)

Figure 2 illustrates that the more severe the complications are, the greater the cost burden is. The figure relates the costs to the average healthcare spend per person living in Bahrain, which was retrieved from the WHO global health expenditure atlas and was used for comparative purposes [29].

**Fig. 3:**
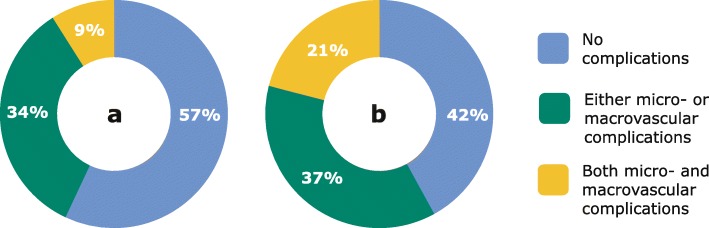
Relative distribution of patients (**a**) and diabetes-attributable costs (**b**) according to complication status

Figure 3 demonstrates the relative distribution of total T2D costs according to complication status and the number of patients. The annual overall treatment cost of T2D for each patient was almost three times more than for a person without T2D [29] (1162 versus 372 BHD (3084 versus 987 USD)). The annual treatment costs of T2D increased progressively with the severity of the complications – exceeding seven times more than for people without T2D and three times more than for people with T2D without complications. It is evident that 9% of people with T2D account for 21% of the treatment costs due to costly diabetes-related complications.

### Healthcare costs attributable to T2D in Bahrain

**Fig. 4:**
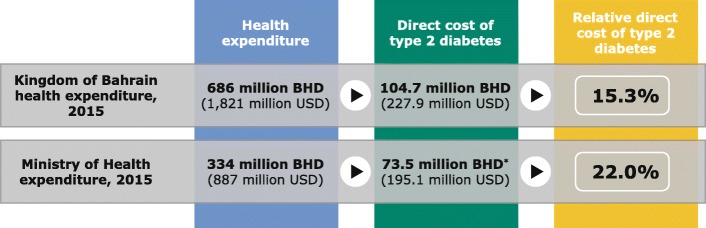
Direct medical costs of T2D from this study compared with total health and Ministry of Health expenditure in Bahrain, 2015

Figure 4 illustrates the extrapolated direct medical costs of T2D in Bahrain compared with total health expenditure and MOH expenditure in Bahrain in 2015. According to the Bahrain Pharmaceuticals and Healthcare Report, the total health expenditure in Bahrain for 2015 was 686 million BHD, equivalent to 1.821 billion USD [30]. Thus, based on this study, the direct medical costs of T2D constituted 15.3% of the total health expenditure. According to the MOH, the healthcare budget was 334 million BHD (887 million USD) in 2015 [31]. Furthermore, general government health expenditure as a proportion of total health expenditure was 70.2% [32]. Thus, the direct medical costs attributable to T2D accounted for 22.0% of the total MOH expenditure (Fig. 4). Furthermore, the direct medical costs constituted 0.89% of GDP in 2015.

### Indirect costs attributable to T2D in Bahrain

Based on information collected through the participants’ questionnaire, the average annual absenteeism due to T2D for each patient was 0.55. As the average wage in Bahrain is 24.9 BHD (66.1 USD) per day [26], the total indirect costs due to absenteeism were approximately 1.23 million BHD (3.26 million USD).

The absolute and relative distribution of the direct medical costs and indirect costs is shown in Table 6 and illustrates that direct medical costs account for the large majority of the total costs [33].

**Table 6:** Absolute and relative distribution of direct medical and indirect costs attributable to T2D in Bahrain

	Million BHD	Million USD	% of total cost	% of GDP [33]
Direct medical costs	104.7O	277.89	98.8%	0.89%
Indirect costs	1.23	3.26	1.2%	0.01%
Total costs	105.93	281.15	100%	0.9%

Bahrain GDP in 2015: 31.13 billion USD [33]

## Discussion

This study is the first to document the healthcare costs of T2D in Bahrain. The direct medical costs of T2D and its related complications were 104.7 million BHD (277.9 million USD) in 2015. The serious financial burden of diabetes is obvious, as people with diabetes require 2–3 times the health resources of people without diabetes [34].

This study confirms that the direct health expenditure for a person with T2D was 3.1 times higher than the average costs for a person without T2D. Furthermore, it clearly illustrates that the direct medical costs attributable to T2D increase with the presence of diabetes-related complications. These findings concur with results from similar studies in other countries that show that diabetes-related complications drive diabetes-related healthcare costs [12, 13, 15, 35–37]. Only a relatively small fraction of the participants in this study had developed both micro- and macrovascular complications. This may be due to the fact that very advanced cases of T2D are referred to specialised secondary healthcare diabetes clinics and thus would not have been recruited to this study. However, even though the group with both micro- and macrovascular complications was relatively small, it accounted for a large proportion of the total T2D expenditure. The primary factor driving increased costs for complications is the requirement for hospitalisation [15, 36, 37]. This shows that hospital admission is by far the most expensive single resource, consuming the largest share of the healthcare costs used to treat T2D, even though a relatively small proportion of patients use this resource. In other words, 20% of the patients consumed 60% of the direct medical costs attributable to T2D due to admissions. The proportion of admission costs is significantly higher than documented in other countries where it is on average 30% [12, 13, 15, 35–37]. This may indicate that there is an opportunity to increase secondary prevention in Bahrain and prevent or delay the onset of serious and expensive T2D-related complications.

The risk of developing serious and expensive complications increases with diabetes duration and higher HbA_1c_ [9, 38]. Studies have shown that blood glucose control is often poor in countries in the MENA region [19, 39–41]. A chi-squared test revealed that in this study the number and severity of complications increased with higher HbA_1c_ (*p* < 0.05). Since blood glucose is an important factor in the development of complications [9], there is great benefit in lowering HbA_1c_ to achieve the official HbA_1c_ treatment target [9, 28, 42]. In this study, approximately one-third of patients had an HbA_1c_ above 8%, which the IDF’s guidelines describe as “unacceptably high” [42] and which is the point at which the risk of complications increases significantly [9]. An additional beneficial approach is to increase attempts to detect and diagnose people with T2D early, in order to initiate optimal treatment during the asymptomatic phase of the condition, to reduce the risk of complications as the condition progresses [43]. Harris et al. documented that a relatively long asymptomatic period of 9–12 years is often the case with T2D [44]. The IDF estimates that up to 49% of people with T2D in the MENA region remain undiagnosed and that the proportion of people with undiagnosed T2D in Bahrain is 38% [2].

For the majority of people with T2D, the primary source of care is their GP, as was the case in this study. However, due to the complexity of managing T2D, primary care providers often maintain patients at suboptimal glycaemic levels, even though there is a willingness to intensify treatment [40, 41, 45]. In this study, just half of the patients achieved the recommended HbA_1c_ target. This suggests that there is a likely advantage to empowering GPs to improve treatment quality [41, 45].

Bommer et al., based on epidemiologic and economic data from 184 countries, estimated that the global cost of diabetes in 2015 was 1.31 trillion USD [46], which was 1.8% of global GDP. They noted that indirect costs accounted for 34.7% of the total burden [46]. They found that the direct medical costs of diabetes represented 0.84% of GDP in the MENA region, with total costs, including indirect costs, accounting for 1.3% of the region’s GDP. Since T2D makes up the vast majority of diabetes cases worldwide [2], these figures can be taken to be largely representative of T2D. This concurs with our finding of the direct medical costs of T2D representing 0.89% of Bahrain’s GDP.

This study has several limitations, which may have contributed to underestimating the treatment cost of T2D. Firstly, people with T2D who were not associated with any clinics, or very advanced cases of T2D referred to secondary clinics, were not included in this study. Secondly, the diabetes prevalence used in this study is based on a survey from 2007 [20]. Other studies indicate that the diabetes prevalence in Bahrain may be substantially underestimated [47]. If the diabetes burden in Bahrain is expected to follow the same development as in other countries, it would have increased since then. Thirdly, treatment costs are expected to rise with increasing age [48], and few patients above the age of 60 were included in this study compared with the survey [20]. Non-medical costs, such as transportation, were not included in this study. It was assumed that in a small country such as Bahrain, transportation would represent an insignificant cost factor, due to an infrastructure where all health centres are located within a 20-min drive of all residents’ homes and of a main hospital.

The human capital approach may misestimate the productivity loss by excluding unpaid work, applying an average salary which varies depending on the type of profession, not accounting for presenteeism (decreased productivity due to ill health while at work) and not including productivity loss due to mortality.

We therefore assume that the estimates illustrated by this study are conservative. However, it is our hope that the challenges of the T2D burden in Bahrain are clear, and that the results will guide decisions on future healthcare investments in T2D.

## Conclusion

In conclusion, this study is the first of its kind to document the health-economic aspects of T2D in Bahrain. It shows that the costs of T2D and its complications constitute an enormous burden to Bahrain. This is not sustainable in the long run, and focusing on secondary prevention by optimising diabetes management and control, thereby reducing or eliminating the risk of disabling and expensive complications, has the greatest potential to counteract this.

## Supplementary information

**Additional file 1.** Diabetes impact in Bahrain questionnaire.

## Data Availability

The datasets used and/or analysed during the current study are available from the corresponding author on reasonable request. The patient questionnaire is included as supplementary material.

## Abbreviations

BHD: Bahraini dinar
GCC: Gulf Cooperation Council
IDF: International Diabetes Federation
MENA: Middle East and North Africa
MOH: Ministry of Health
T2D: Type 2 diabetes
USD: United States dollar

## Footnotes

1 Currency converter: 1 BHD = 2.6542 USD on 31 December 2015. https://www.currency-converter.org.uk/currency-rates/historical/table/BHD-USD.html.
