# Supplementary material for: Type 2 diabetes and healthcare resource utilisation in the Kingdom of Bahrain
Source: BMC Health Serv Res. 2019 Dec 5;19:939. doi: 10.1186/s12913-019-4795-5 (PMC6896470; doi:10.1186/s12913-019-4795-5)
Supplement: Supplementary file 1 — Additional file 1. Diabetes impact in Bahrain questionnaire. [file 12913_2019_4795_MOESM1_ESM.pdf]

KINGDOM OF BAHRAIN
MINISTRY OF HEALTH

Together for Better Life

DIABETES IMPACT IN BAHRAIN
DIABETIC SUBJECT

DEMOGRAPHIC DATA

Patient File Number: ☐☐☐☐☐☐☐ **Serial Number:** ☐☐☐☐

First *Middle* *Last*

Name: ____________________

CPR NO: ☐☐☐☐☐☐☐☐☐

DD *MM* *YYYY*

Date of Birth: ☐☐☐

Gender: ❑ Male ❑ Female

Contact Number: ______ / ______ / ______ /

Educational Level:

❑ Illiterate

❑ Primary

❑ Intermediate

❑ Secondary

- ❑ Higher Education

Smoking: ❑ Smoker ❑ Non-smoker ❑ Ex-smoker

Management:

- ❑ ACEI
- ❑ ARBs
- ❑ Aspirin
- ❑ Statins
- ❑ Fibrates
- ❑ Anti-HTN Drugs

Smoking Advice Given	❑ Yes	❑ No
Low Protein Diet	❑ Yes	❑ No

DIABETES HISTORY:

Family History (First and Distant Relatives)

- ❑ DM
- ❑ HTN
- ❑ Hyperlipidemia
- ❑ Chronic Disease
- ❑ Renal Disease

TYPE OF DIABETES:

- ❑ Type 1 Diabetes
- ❑ Type 2 Diabetes
- ❑ IGT
- ❑ Gestational diabetes
- ❑ Other types (Specify : _______)

DURATION: __________ Years

TREATMENT:

Insulin			
**Type**	**Morning**	**Noon**	**Evening**
Regular	units	units	units
NPH	units	units	units
Mixed	units	units	units
Other	units	units	units

Hypoglycemic Agents		
**Type**	**Dose**	**Frequency**
1.		
2.		
3.		
4.		

COMPLICATIONS:

❑ DKA per year _______ ❑ Hypoglycemia ______ per year

	**Yes**	**No**	**Not done**
- Neuropathy	❑	❑	❑
- Retinopathy	❑	❑	❑
- Nephropathy	❑	❑	❑

- Vasculopathy: **Yes** ❑ **No** ❑

1.CVA **Yes** ❑ **No** ❑

2.IHD **Yes** ❑ **No** ❑

Chest Pain **Yes**❑ **No** ❑

ECG **Normal** ❑ **Abnormal** ❑

3. PVD **Yes** ❑ **No** ❑

-Foot ulcer **Yes** ❑ **No** ❑

-Cataract **Yes** ❑ **No** ❑

-Dental Problem **Yes** ❑ **No** ❑

-Impotence **Yes** ❑ **No** ❑

SOCIAL IMPACT (ADULTS):

DIABETES IS:

Yes	No	
❑	❑	Serious Disease
❑	❑	Inherited Disease
❑	❑	Socially Restricting Disease
❑	❑	Cause Work Loss

Work Difficulties Per Year	Number	Duration Per Day	Total Days
Work Permission			
Sick Leave			
Salary Deduction			

HANDICAP:

1. **Visual**: ❑ Decrease Vision ❑ Blindness
2. **Ambulatory**: ❑ Amputation ❑ Stroke
3. **Mental**: ❑ Yes ❑ No

MEDICAL SERVICE:

1. **Who?**
   - ❑ None
   - ❑ General Practitioner
   - ❑ Internist
   - ❑ Endocrinologist

2. What? ❑ Outpatient _____/year , Lab tests _____/year

❑ Admission _____/year, Duration _____/total days/year

3. Where? ❑ Public Hospital

❑ Primary Care Center

❑ Private Hospital

❑ Private Clinic

FINANCIAL IMPACT:

Do You Pay for Diabetes:

❑ Yes (How much BD/year) ❑ No

Do You Have Health Insurance:

❑ Yes (Specify:) ❑ No

Type of Payment	MOH	others	Personal	Insurance
Drugs				
Lab test				
Glucometer strips				
Doctor visits				
Admissions				
Other				

N.B.: others: ❑ALBA ❑BAPCO ❑G.A clinic ❑BRAMCO ❑BDF Hospital

PROCEDURES RELATED:

Procedures	Frequency	Place
Cardiac Cath		
Laser		
Cataract Surgery		
Dialysis		

BLOOD SUGAR MONITORING:

Procedures	Frequency/yr	Place
FBS		
RBS		
OGTT		
HbA1c		

PHYSICAL EXAMINATION & LABORATORY RESULTS

Vital & Anthropometric Data		
**Parameter**		**DATE:**
**BP & Weight**	BP sitting (mmHg)	
	Weight (kg)	
	H eight (cm)	
	Waist (cm)	
	W/H ratio	
	BMI (m^2)	
**Foot Examination**	Pluses(DP,PT,RAD)	
	Vibration (128Hz)	
	Touch(10 g monofilament)	
**Urine**	Proteins (dipstick)	
**Heamatological markers**	HB<br>CRP<br>B12<br>Homosystine	
**Gluco-markers**	FBS (mmol/l)	
	HbA1c (%)	
**Renal Profile**	Creatinine (mmol/l)/eGFR if Cr > 85	
	Ca +2	
	P04-	
	Vit D level	

**Lipid Profile**	Triglycerides (mmol/l)	
	Cholesterol (mmol/l)	
	HDL (mmol/l)	
	LDL (mmol/l)	
**Hormonal Profile If indicated**	FSH,LH	
	Testosterone	
	PR	
	TSH<br>S.Insulin	

Parameter	First Visit<br>Date:	Second Visit<br>Date:
1. ACR (if protein dipstick negative)		
2. PCR (if protein dipstick positive)		
eGFR<br>(If 1 OR 2 Positive)		
